# Developing a Minimally Structured Mathematical Model of Cancer Treatment with Oncolytic Viruses and Dendritic Cell Injections

**DOI:** 10.1155/2018/8760371

**Published:** 2018-10-30

**Authors:** Jana L. Gevertz, Joanna R. Wares

**Affiliations:** ^1^Department of Mathematics & Statistics, The College of New Jersey, Ewing, New Jersey, USA; ^2^Department of Mathematics and Computer Science, University of Richmond, Richmond, Virginia, USA

## Abstract

Mathematical models of biological systems must strike a balance between being sufficiently complex to capture important biological features, while being simple enough that they remain tractable through analysis or simulation. In this work, we rigorously explore how to balance these competing interests when modeling murine melanoma treatment with oncolytic viruses and dendritic cell injections. Previously, we developed a system of six ordinary differential equations containing fourteen parameters that well describes experimental data on the efficacy of these treatments. Here, we explore whether this previously developed model is the minimal model needed to accurately describe the data. Using a variety of techniques, including sensitivity analyses and a parameter sloppiness analysis, we find that our model can be reduced by one variable and three parameters and still give excellent fits to the data. We also argue that our model is not too simple to capture the dynamics of the data, and that the original and minimal models make similar predictions about the efficacy and robustness of protocols not considered in experiments. Reducing the model to its minimal form allows us to increase the tractability of the system in the face of parametric uncertainty.

## 1. Introduction

For many solid tumors, the most utilized cancer treatments are surgery, chemotherapy, and radiotherapy [1]. While this approach can effectively reduce tumor burden in the short term, long-term recurrence is the norm. This failure of conventional treatment modalities has spurred efforts to design novel cancer therapeutics.

One emerging treatment modality is oncolytic virotherapy. This technique involves targeting cancer cells using oncolytic viruses (OVs), standard viruses genetically engineered to replicate selectively in cancer cells. OV replication within a cancer cell creates a viral burden too large for the cell to support, which eventually causes the infected cancer cell to lyse [2]. The OVs that get released from the lysed cancer cell are then free to infect additional cancer cells. The lysing effects of OVs, while powerful, are also transient. In a clinical setting, OVs have generally proven to be insufficient to fully and permanently eradicate a solid tumor mass [2].

Another treatment modality receiving attention is gene therapy, defined as the introduction of genes of interest into cancer cells with therapeutic intent [3]. Gene therapy has been attempted using genes that mediate the release of cytokines, tumor suppressor genes, and apoptosis-related genes, to name a few. Independent of the gene of interest, this modality requires a vector that can efficiently deliver, and uniformly distribute, the gene product to solid tumors [4]. Oncolytic viruses can be used as such a gene-delivery vector.

The ability of oncolytic virotherapy to induce tumor cell lysis and to stimulate an antitumor immune response in a preclinical setting has led to a number of clinical trials for different tumor types. As of 2016, twenty different viruses have been studied as candidates for oncolytic virotherapy, and new candidate viruses continue to be studied [5]. In 2015, the U.S. Food and Drug Administration approved the first oncolytic virus therapy, T-VEC (Imlygic™), for the treatment of advanced melanoma [5]. A number of clinical trials using this and other oncolytic viruses are underway in both solid and liquid tumors, and while they tend to be incredibly well tolerated, to date efficacy has been inferior to other available therapies [5, 6]. That said, researchers continue to actively study the anticancer effects of oncolytic virotherapy, both as a drug to be used in combination with other modalities, and as a potential cancer vaccine [5, 6].

In the present work, we focus on preclinical data and model the use of OVs (the adenovirus (Ad) in particular) to deliver genes that boost the immune system's ability to identify, target, and kill cancer cells. The transgenes of interest in this study are 4-1BB ligand (4-1BBL) and interleukin (IL)-12. 4-1BB is a costimulatory member of the tumor-necrosis factor receptor superfamily that is expressed on activated CD4+ and CD8+ T cells [7]. The binding of 4-1BB to its ligand, 4-1BBL, promotes the outgrowth of type-1 T helper cells and cytolytic effector T cells [4]. IL-12 is a cytokine that strongly stimulates the differentiation of naïve CD4+ T cells to type-1 T helper cells. IL-12 has been determined to be one of the most efficient antitumor cytokines in experimental animal models [8]. For conciseness, we refer to oncolytic adenoviruses concurrently acting as a vector for both the 4-1BBL and IL-12 transgenes as Ad/4-1BBL/IL-12.

Recent preclinical work of Huang et al. has shown that Ad/4-1BBL/IL-12 can cause tumor regression in a mouse model of melanoma [4]. This debulking is a consequence of both tumor cell lysis, as well as immune system stimulation resulting from the local release of 4-1BBL and IL-12. Ad/4-1BBL/IL-12 can also be combined with intratumorally injected dendritic cell (DC) vaccines, resulting in a greater antitumor response than elicited by either treatment alone [4]. DCs are immune cells that present antigens to other cells of the immune system. Antigen presentation triggers an adaptive immune response that results in the immune system actively seeking out cells expressing the presented cancer antigen [9]. Huang et al. developed DC vaccines by harvesting DCs from the bone marrow of tumor-bearing mice, and exposing them ex vivo to tumor-associated antigens until maturation [4].

Given the combined effectiveness of Ad/4-1BBL/IL-12 and DC injections, it is natural to ask in what order, and at what dose, one should administer these therapeutics to elicit the maximal antitumor response. This is an experimentally time-consuming and costly question to address. Mathematical-modeling techniques can help answer questions about complex biological systems without the associated experimental costs [10]. Differential equation models (frequently paired with experimental data) have been successfully used to improve treatment protocols involving oncolytic viruses [11–16]. Previously, we hierarchically developed and fit a mathematical model to the experimental data in Huang et al. [4]. This system of ordinary differential equations, involving six variables and fourteen non-initial-condition parameters, was shown to well describe the dynamics of OVs enhanced with one or more immunostimulatory molecules (4-1BBL, IL-12, or both), DC injections, and DC injections coupled with Ad/4-1BBL/IL-12 [17–19]. Note that for each treatment protocol, the model was fit to the *average* tumor volume data (averaged over the 8-9 mice in the treatment cohort, with some consideration of the standard deviation in the data).

Using the best-fit parameters obtained from the hierarchical fitting to the average, we previously discovered that administering three doses of OVs followed by three doses of DCs (OV-OV-OV-DC-DC-DC) is the optimal drug ordering [18] when constrained to considering the drug dosing and spacing used by Huang et al. [4]. Further analysis, however, led us to doubt the robustness of this prediction. First, we found that the doses of Ad/4-1BBL/IL-12 and DC used in Huang et al.'s experiments [4] were near a bifurcation point; that is, slightly altering the dose or sequence could drastically change the efficacy of the protocol [18]. A full-scale robustness analysis of optimal protocols using the Virtual Expansion of Populations for Analyzing Robustness of Therapies (VEPART) procedure confirmed that our originally predicted optimal strategy is fragile [19].

The fragile nature of the optimal protocol raises doubts about whether it will actually be effective in individual mice in the experimental population, as individual mice generally have dynamics that deviate from the average. And, in the era of personalized medicine [20, 21], there is a strong emphasis on tailoring treatment protocols to individual patients. However, the difficulty of collecting and analyzing patient-specific data, especially in the face of intratumor and temporal heterogeneity, makes personalizing therapy a real challenge [22]. Even individualizing a mathematical model is a highly nontrivial task, as it often requires finding the best-fit parameters in a high-dimensional parameter space, given a very limited amount of data about the individual. Therefore, before we can explore the challenging question of personalizing therapy using our model, we ask the following questions: does our mathematical model require all six variables and fourteen parameters to adequately describe the data? Or, can we simplify the structure of the model (number of variables and parameters) in order to make the model more amenable to personalization while retaining the goodness of fit to the experimental data? These are the questions that we answer in this work.

This paper is organized as follows. First, we briefly introduce the reader to the previously developed mathematical model of tumor growth and treatment with Ad/4-1BBL/IL-12 and DCs [17, 18]. Second, we introduce a collection of methods that we employ to test whether our original model is minimal in its structure. Third, we argue that the original model is not of minimal structure, supported by evidence from parameter 95% credible intervals, local and global sensitivity analyses of parameters, and a soft/stiff parameter analysis. This leads us to propose a minimal model that contains five variables (one less than the original model) and eleven non-initial condition parameters (three less than the original model). Next, we show that this minimal model fits the experimental data as well as the original model, and we further argue that the model is not too simple for describing Huang et al.'s experimental data [4]. We conclude by showing that the minimal and original models make qualitatively similar predictions about the efficacy and robustness of treatment protocols not considered in our experimental dataset, which serves to further validate the sufficiency of the minimal model.

## 2. Methods

We begin by introducing the previously developed mathematical model that describes tumor-immune dynamics subject to treatment with either OVs, DCs, or both [17, 18]. We then expound upon the variety of techniques that we utilized to address whether the model is minimally structured, given the experimental data it was designed to fit.

### 2.1. Original Mathematical Model

Our original model contained the following six ordinary differential equations:

(1) $$\begin{array}{c} \frac{dU}{dt}=rU-\beta \frac{UV}{N}-\left(\kappa_{0}+c_{\text{kill}}I\right)\frac{UT}{N},\;U\left(0\right)=U_{0}, \end{array}$$

(2) $$\begin{array}{c} \frac{dI}{dt}=\beta \frac{UV}{N}-\delta_{I}I-\left(\kappa_{0}+c_{\text{kill}}I\right)\frac{IT}{N},\;I\left(0\right)=0, \end{array}$$

(3) $$\begin{array}{c} \frac{dV}{dt}=u_{\text{V}}\left(t\right)+\alpha \delta_{\text{I}}I-\delta_{\text{V}}V,\;V\left(0\right)=0, \end{array}$$

(4) $$\begin{array}{c} \frac{dT}{dt}=c_{\text{T}}I+\chi_{\text{A}}A+\chi_{\text{D}}D-\delta_{\text{T}}T,\;T\left(0\right)=0,\; \end{array}$$

(5) $$\begin{array}{c} \frac{dA}{dt}=c_{\text{A}}I-\delta_{\text{A}}A,\;A\left(0\right)=0, \end{array}$$

(6) $$\begin{array}{c} \frac{dD}{dt}=u_{\text{D}}\left(t\right)-\delta_{\text{D}}D,\;D\left(0\right)=0. \end{array}$$

When all parameters and time-varying terms are positive, this model captures the effects of tumor growth and response to treatment with Ad/4-1BBL/IL-12 and DCs. By allowing various parameters and time-varying terms to be identically zero, other treatment protocols tested in Huang et al. can also be described (e.g., adenovirus that only mediates the release of 4-1BBL) [4]. These other models are defined more explicitly after the description of the full model.

In Equation (1), uninfected tumor cell volume, *U*, grows exponentially (at a rate *r*), and upon being infected by an oncolytic adenovirus, *V*, converts to infected cancer cell volume, *I*, at a density-dependent rate (*βUV*/*N*), where *N* is the total volume of cells (tumor cells and tumor-targeting T cells, *T*). The tumor-targeting T cells indiscriminately kill both uninfected and infected tumor cells, with the rate of killing depending on the amount of IL-12 and 4-1BBL production (modeled through *I* in the term (*κ*_0_+*c*_kill_*I*)).

In Equation (2), newly infected tumor cell volume is accumulated at a rate of *βUV*/*N*. The infected cells, *I*, are lysed by the virus or other mechanisms (at a rate of *δ*_I_), thus acting as a source term for the virus by releasing free virions into the tissue space (with *α* virions released on average per cell, seen in Equation (3)). We again see the tumor-targeting T cells in action, killing *I* (modeled in the term (*κ*_0_+*c*_kill_*I*)).

In Equation (3), treatment with OVs is represented with the time-dependent term *u*_V_(*t*), determined by drug delivery and dosing schedules of interest. Due to lysis of *I*, *α* virions are released on average per cell lysed, as discussed earlier. Virions are not impacted by the T cell population—any loss of virions is due to natural decay, *δ*_V_.

Equations (4) and (5) describe how the population of T cells, *T*, and naïve T cells, *A*, change in time. The activation and recruitment of tumor-targeting T cells can happen in three ways: (1) through stimulation of the naïve T cell pool, which at rate *χ*_A_ can asymmetrically divide to give rise to tumor-targeting T cells, due to increased IL-12 (and modeled as proportional to *I*, at a rate of *c*_A_, as infected cells are the ones to release IL-12); (2) through stimulation of cytotoxic T cells due to increased 4-1BBL (also modeled as proportional to *I*, at a rate of *c*_T_); and (3) through production of T cells due to the externally primed dendritic cells, *D*, (at a rate of *χ*_D_). T cells and naïve T cells also experience natural death (at rates of *δ*_T_ and *δ*_A_, respectively).

Equation (6) describes how the population of injected dendritic cells changes over time. The time-dependent term *u*_D_(*t*) represents the treatment with DCs, determined by the drug delivery and dosing schedule of interest. Dendritic cells decay at a rate of *δ*_D_.

The data from Huang et al. was measured as tumor volume versus time for a variety of treatment protocols, averaged over 8-9 mice per protocol [4]. The treatment protocols increased in complexity, and therefore, our full model was designed and validated hierarchically against each dataset as follows:

- *Model 1*: *No OV or DC treatment*. Under no treatment, tumor data are modeled using Equation (1) only, with all parameters other than *r* set to 0.
- *Model 2*: *Treatment with OVs that replicate and lyse, but do not mediate the release of cytokines and costimulatory molecules*. Tumor data under this treatment are modeled using Equations (1)–(3) with *κ*_0_ and *c*_kill_ set to 0.
- *Model 3*: *Treatment with OVs that lyse tumor cells and*
  - *mediate the release of 4-1BBL (Ad/4-1BBL)*. Tumor data under this treatment are modeled using Equations (1)–(4) with *χ*_A_ and *χ*_D_ parameters in Equation (4) set to 0.
  - *mediate the release of IL-12 (Ad/IL-12)*. Tumor data under this treatment are modeled using Equations (1)–(5) with *c*_T_ and *χ*_D_ parameters in Equation (4) set to 0.
  - *mediate the release of both IL-12 and 4-1BBL (Ad/4-1BBL/IL-12)*. Tumor data under this treatment are modeled using Equations (1)–(5) with *χ*_D_ parameter in Equation (4) set to 0.
- *Model 4*: *Treatment with DCs only*. Tumor data under this treatment are modeled using only Equations (1), (4), and (6). Equation (4) has *c*_T_ and *χ*_A_ parameters set to 0 in this case, and Equation (1) has *β* set to 0.
- *Model 5*: *Treatment with Ad/4-1BBL/IL-12 and DCs*. This is modeled using the entirety of the system in Equations (1)–(6).

The experimental data increase from simple to more complex, allowing us to fit the model parameters in a hierarchical fashion. For instance, using Model 1, we fit the tumor growth rate *r* to the control data in which the tumors grew without treatment. The best-fit value of *r* was then used in subsequent versions of the model. Using previously fit parameters allowed us to reduce the dimension of parameter space at each step of the model development and fitting process. More details on the hierarchical development of the model can be found in our previous studies [17–19].

### 2.2. 95% Credible Intervals and Local Sensitivity Analysis

In our original works [17, 18], a single best-fit value was determined for each parameter in system (1)–(6). In our later work [19], we expanded our understanding of the best-fit parameter values by identifying the potential distribution for each parameter. We did this by creating one thousand bootstrap replicates [23] from each of our experimental datasets (control, Ad only, Ad/4-1BBL/IL-12, etc.,) [19]. Each bootstrap replicate was created by sampling the *N* mice in the original experimental dataset with replacement. The best-fit parameter values were found for each bootstrap replicate, and these were used to estimate the posterior marginal distribution on each fit parameter. In other words, each distribution we approximate is the distribution of the sample average of a parameter value, for one thousand populations of size *N*. The interval in which we can be 95% certain that the true value of the parameter is found, the 95% credible interval, was then calculated from these approximated distributions. This is done by excluding the values that fall in the extreme tails of the distribution (2.5% of values in each tail). In this work, we identify poorly constrained parameters (that may not be needed in a minimal model) as those with very large 95% credible intervals.

We have previously performed a local sensitivity analysis on the full system in Equations (1)–(6), along with the submodels that describe the simpler treatment protocols detailed above [19]. The parameters we focus on are the ones whose values could not be readily ascertained from experiments, as detailed in [18]. For each submodel, our local sensitivity analysis entails performing an exhaustive search about the best-fit parameters and identifying all parameter sets that give a fit within 10% of the optimal fit.

The optimal fit is defined as the set of parameters that minimize the goodness-of-fit metric *ζ* [19]:

(7) $$\begin{array}{c} \zeta =\underset{t}{\sum }v_{t}\frac{{\left(v_{t}^{\text{model}}-v_{t}\right)}^{2}}{\sigma_{t}^{2}}, \end{array}$$

where *v*_t_ is the average experimental tumor volume at day *t*, *v*_*t*_^model^ is the tumor volume at day *t* predicted by the submodel under consideration, and *σ*_*t*_^2^ is the variance in the experimental tumor volume at day *t*. The fractional term in Equation (7) is a dimensionless measure of the error in which the sum of the square error is divided by the variance in the experimental data. In this way, we require better fits to the average volume when the variance is small, in accordance with the principle of maximum likelihood estimation [24]. However, because instrumentation error in volume measurements is independent of tumor volume, calipers are imprecise for smaller tumor sizes [25, 26]. For this reason, we weigh the dimensionless term in Equation (7) by the average tumor volume, as this does not artificially bias the algorithm to fit well at small tumor sizes at the expense of fitting well over a majority of the data points [19].

In this work, we use these data to identify parameters that the model is highly insensitive to (parameters that can vary widely without affecting the goodness of fit of the model). We then consider fixing each of those parameters or even restructuring the equations to remove such parameters all together.

### 2.3. Global Sensitivity Analysis

We next take a more holistic view of parameter space by performing a global sensitivity analysis using the Sobol method [27]. To do this, we view parameter space as our input space, and the tumor volume as predicted by the corresponding DE submodel in Equations (1)–(6) as the output space. The Sobol method allows us to determine how much of the total output variance is due to each individual parameter. If the Sobol Index corresponding to a particular parameter (defined below) is small, the model dynamics are insensitive to varying that particular parameter.

To use the Sobol method, we first determine a realistic domain for each parameter in the model. Define the vector *p* such that each element of the vector corresponds to a parameter in our model, specified in Equations (1)–(6):

(8) $$\begin{array}{c} p=\left[r\;\beta \;\kappa_{0}\;c_{\text{kill}}\;\delta_{\text{I}}\;\alpha \;\delta_{\text{V}}\;c_{\text{T}}\;\chi_{\text{A}}\;\chi_{\text{D}}\;\delta_{\text{T}}\;c_{\text{A}}\;\delta_{\text{A}}\;\delta_{\text{D}}\right]. \end{array}$$

The full set of choices for each element of the vector *p* defines the 14 dimensional parameter space *P*; though in all of our analyses, we only consider a subset of this vector. We assume a uniform distribution for each parameter in the parameter space, where the minimum and maximum value for each parameter is determined from its 95% credible interval.

Here, we define the total output space as all possible values of *y*=*f*(*p*), where for a particular choice of parameters *p* ∈ *P*, we define

(9) $$\begin{array}{c} f\left(p\right)={\int^{}}_{0}^{t_{\text{final}}}\left[U\left(t\right)+I\left(t\right)\right]dt, \end{array}$$

where *U*(*t*) and *I*(*t*) are elements of the solution to the original model (1)–(6), for the particular choice of parameters *p* with time *t*=0 to *t*=*t*_final_, here 30 days.

In order to determine how the output varies with each parameter, we consider how much each parameter contributes to the total variance of the output space [27, 28], var{*y*}. First we calculate the total variance of *y* over the full parameter space, var{*y*}. If possible, we would next calculate the variance of *y*, given the true value of $p_{i}={\bar{p}}_{i}$, $\mathrm{var}\left\{y\left|{\bar{p}}_{i}\right.\right\}$, to determine how much variance is lost due to *p*_*i*_ being fixed. However, we do not know the true value of *p*_*i*_. Instead, we calculate the first-order sensitivity index (Sobol Index) for each of the *i* parameters, *S*_*i*_, by calculating the expected value of *y* over all other parameters conditioned on each value of *p*_*i*_: *h*(*p*_*i*_)=*E*[*y*|*p*_*i*_]. We then compute the variance of the conditional expectation: var[*h*(*p*_*i*_)].

*S*_*i*_ is then defined as the amount that the parameter *p*_*i*_ contributes to the total variance, normalized by the total variance:

(10) $$\begin{array}{c} S_{i}=\frac{\mathrm{var}\left[h\left(p_{i}\right)\right]}{\mathrm{var}\left[y\right]}=\frac{\mathrm{var}\left[h\left(p_{i}\right)\right]}{\int_{P}f\left(P\right)\;dP}, \end{array}$$

where, as previously stated, the range of values considered for each parameter *p*_*i*_ is determined by its associated 95% credible interval.

Sobol's key insight involves using an ANOVA decomposition to calculate the above variances [27, 28]. This allows for practical numerical simulations that employ Monte Carlo simulations to calculate the Sobol Indices [27]. In this study, we utilized an available MATLAB toolbox, GSAT, to calculate all of the reported Sobol Indices using the FAST method and Sobol distributions for sampling. (https://www.mathworks.com/matlabcentral/fileexchange/40759-global-sensitivity-analysis-toolbox). When Sobol's method determines that varying a parameter over its domain causes *y* to change minimally, *S*_*i*_ will be small. Because such a *p*_*i*_ does not contribute much to the overall variance of the output, the model is considered insensitive to the value of that parameter. On the contrary, when varying a parameter over its domain causes *y* to greatly vary, *S*_*i*_ will be large. A model is sensitive to parameters *p*_*i*_ corresponding to large *S*_*i*_ values.

### 2.4. Parameter Sloppiness Analysis

It is well established that the quantitative behavior of multiparametric biological models is much more sensitive to changes in certain combinations of parameters than to others, a phenomenon known as “sloppiness” [29]. Herein, we will use an established methodology (see [29, 30] for more details) to identify parametric combinations to which the model is highly sensitive (i.e., stiff directions) and combinations to which the model is highly insensitive (i.e., soft directions). This is done by performing an eigenvector decomposition of the Hessian matrix corresponding to our cost function in Equation (7). Large eigenvalues correspond to stiff eigendirections, whereas small eigenvalues correspond to the soft eigendirections [29, 30].

**Figure 1:**
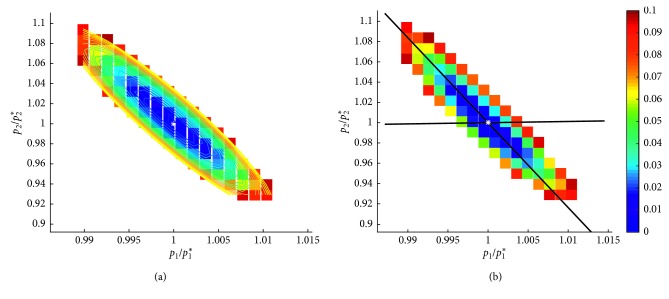
An example illustrating the ellipsoidal nature of the cost function near the best-fit parameters for Equation (10). Note that parameters on each axis are scaled by the optimal value, indicated with *a*^*∗*^. Only parameters where the cost is less than 0.1 (deviation from optimal value of *ζ* is 10% or less) are shown. (a) Also shows the contours of the best-fit quadratic surface to this data. (b) Also shows the eigenvectors of the associated Hessian matrix. The near-vertical eigenvector (which appears less vertical than it actually is due to axes scaling) has *λ*_1_=11.9947 with ${\vec{v}}_{1}=\left[0.11857,-0.99295\right]$, and the near-horizontal eigenvector has *λ*_1_=5686.0 with ${\vec{v}}_{2}=\left[0.99295,0.11857\right]$.

The foundational step of performing a sloppiness analysis is to compute the cost manifold [30]. We do this by identifying all points in parameter space that give a fit within 10% of the optimal (generated for the previously-described local sensitivity analysis). The data are then normalized in two ways. First, the value of each parameter is scaled by the optimal value of that parameter, hence the normalized axes we see in Figure 1. Second, for each parameter set {*p*_*i*_}, we define the cost function *c*({*p*_*i*_}) to be the deviation in the value of *ζ* at that parameter set {*p*_*i*_} compared with the value of *ζ* at the optimal parameter set. As we are only interested in parameters that give a fit within 10% of optimal, each point shown in parameter space in Figure 1 has a *c* value in the range 0 and 0.1. We then find the best-fit quadratic surface describing *c*({*p*_*i*_}) using the polyfit package for MATLAB (https://www.mathworks.com/matlabcentral/fileexchange/34765-polyfitn). Once the best-fit quadratic surface has been obtained (an example of which is visualized in Figure 1), its associated Hessian matrix can be computed. The eigenvalues and eigenvectors of this matrix allow us to identify soft and stiff parameter directions.

We will first employ this analysis to study whether the OV model has any redundant parameters. This means we will compute *c*({*p*_*i*_}) as a function of the T cell parameters (*c*_A_, *c*_T_, *c*_kill_) in Model 3c, which is our model of treatment with Ad/4-1BBL/IL-12 only. We choose to focus on the T cell parameters because our local sensitivity results (see Section 3.1) suggest that the model is most insensitive to these parameters. The second question we will explore is whether the model is too simple to describe the experimental data. To this end, we turn to one of the major simplifying assumptions we made: that tumor growth is exponential in the absence of treatment, and we consider instead a model without any treatment (*u*_v_(*t*)=*u*_D_(*t*)=0  ∀ *t*) in which tumor growth is logistic instead of exponential. In this case, Model 1 is replaced with the model:

(11) $$\begin{array}{c} \frac{dU}{dt}=p_{1}U\left(1-\frac{U}{p_{2}}\right),\;U\left(0\right)=U_{0}, \end{array}$$

and we will be working in (*p*_1_, *p*_2_) parameter space, where *p*_1_ is the tumor growth rate (comparable to *r* in the original Model 1), and *p*_2_ is the tumor-carrying capacity. We perform a parameter sloppiness analysis to assess the carrying capacity's impact on the fit of the model to the data. While there are other intrinsic tumor growth terms that could be considered, and other structural terms in the model we could analyze, we focus on the exponential growth term because prior studies have indicated better fits to cancer growth data using functional forms other than exponential growth [31].

### 2.5. Robustness Analysis

Once we propose a minimal system, we need to validate that this reduced system and the original system in (1)–(6) make qualitatively (and possibly quantitatively) similar predictions. One way to do this is to compare the fits of the original and reduced models with the data. As a further way to validate the proposed minimal system, we will employ the Virtual Expansion of Populations for Analyzing Robustness of Therapies (VEPART) method [19] to see if the original and reduced systems make similar predictions about treatment efficacy in situations for which we do not have experimental data.

The VEPART method is a way to not only predict optimal treatment protocols, but to assess the *robustness* of those protocols. To detail, when a dataset is mathematically modeled, typically the model is fit to the average experimental data (tumor volume at each time point averaged over the *N* mice in the treatment cohort). From a model that well describes the average data, optimization techniques can be utilized to identify the best way to control the tumor, given the drugs under consideration and any constraints on their usage. However, a protocol that is optimal for treating the “average” tumor may or may not be effective in a tumor whose dynamics deviate from the average. If a protocol elicits the same qualitative response in samples that deviate from the average, we call the protocol *robust*. If a protocol results in a qualitatively different treatment response in samples that deviate from the average, the protocol is classified as *fragile* [19].

**Figure 2:**
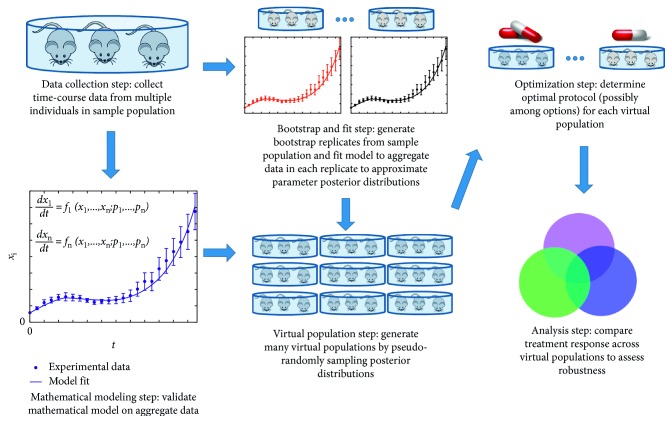
Summary of the virtual expansion of populations for analyzing robustness of therapies (VEPART) method [19].

We have previously undertaken a robust/fragile treatment analysis of system (1)–(6) using the VEPART method [19]. This method, which is summarized in Figure 2, begins with time course data from a sample population, for which a mathematical model is developed and fit to the average of this data. Bootstrapping of the data allows for an estimation of the posterior distribution on each of the fit parameters (in our case, at different stages of the hierarchical model development process). The distributions are then pseudorandomly sampled, only selecting values within the 95% credible interval, and selecting simultaneously fit parameters together to preserve the covariance structure in the data. This procedure results in a full parameterization of the mathematical model, which we call a “virtual population.” We generate 1000 such virtual populations, perform an optimal treatment analysis on each population, and compare treatment response across virtual populations to assess robustness. Full details of the method can be found in [19].

In this work, we will apply the VEPART method to our proposed minimal model and ask whether this model gives qualitatively similar robust/fragile predictions as the original model. If the reduced model indeed gives similar predictions, this provides an additional level of validation that important information was not lost by simplifying our original model to the proposed minimal one.

## 3. Results and Discussion

### 3.1. 95% Credible Intervals and Local Sensitivity Analysis

Here, we explore if any parameters can be fixed or removed entirely from the model without compromising the goodness of fit to the data by considering the following: (1) the 95% credible intervals for each fit parameter and (2) the extent to which a single parameter can deviate from its best-fit value and still give a goodness-of-fit metric (a value of *ζ*) within 10% of the optimal value (Table 1).

If we start by looking at the 95% credible intervals (Table 1), we find that three parameters (*r*, *β*, and *χ*_D_) have very tight credible intervals. Here, tight means that the interval for which we can be 95% certain that the true value of the parameter is found only contains numbers that vary over at most one order of magnitude. On the contrary, the T cell-related parameters (*c*_A_, *c*_T_, and *c*_kill_) have 95% credible intervals containing values that vary over four to five orders of magnitude. This indicates that we have much less certainty about the value of these T cell parameters, and that the model's fit to the data may not heavily depend on their precise value.

**Figure 3:**
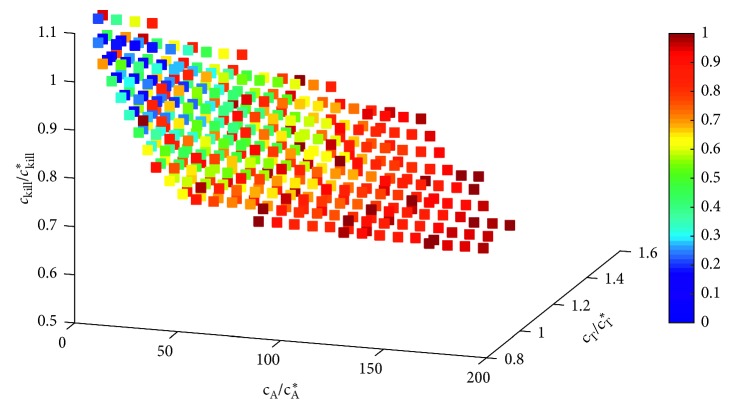
Local sensitivity analysis about optimal parameters in Model 3c (Ad/4-1BBL/IL-12) in *c*_T_ − *c*_A_ − *c*_kill_ space. Parameter values shown on each axis are scaled by the optimal value (indicated with *∗*). Observe how many points with *c*_A_=0 give a fit within 10% of the optimal fit.

To determine how much the fit depends on these parameters, we next consider how much a parameter value can vary from its best-fit value and still give a goodness-of-fit metric *ζ* within 10% of the optimal value. Using the data in Table 1, the *c*_A_ parameter, which represents the IL-12-induced activation rate of naïve T cells, stands out from the other parameters. This parameter can vary by 17,300% from its best-fit value while still ensuring the goodness-of-fit metric *ζ* is within 10% of the optimal value. No other parameters considered come close to being able to deviate this much, with *c*_T_ and *c*_kill_ having the next largest deviations at only 47.2% and 46.3%, respectively. Furthermore, even choosing *c*_A_=0 gives a fit within 10% of the optimal, as shown in the cost function in Figure 3. Combining this local sensitivity result with the very large 95% credible interval for *c*_A_ suggests we can set this parameter to zero in the model. Since the model uses an initial condition of *A*(0)=0, setting *c*_A_=0 does more than just eliminate a parameter from the model—it actually eliminates the entire variable *A* from the model, since the only source term of *A* in Equation (5) comes from the term *c*_A_*I*.

### 3.2. Global Sensitivity Analysis

To further our investigation, we next expand our study of parameter sensitivity from a local one to a global one. In particular, we conducted a Sobol sensitivity analysis on the T cell parameters (*c*_A_, *c*_T_, *c*_kill_) that get fit in the Ad/4-1BBL/IL-12 model (Model 3c). We found that the first-order Sobol indices *S*_param_ are given by *S*_*c*_T__=0.3071, *S*_*c*_kill__=0.2380, and *S*_*c*_A__=0.1359. The larger the Sobol index is for a parameter, the more sensitive the dynamics of the model are to the value of that parameter. We see that *c*_A_ has the smallest Sobol index among the T cell parameters, in spite of the large 95% credible interval used in its calculation.

Thus, the global sensitivity analysis further confirms the local sensitivity analysis: the model appears most insensitive to the choice of *c*_A_. Since we previously showed that *c*_A_=0 is a viable choice for the model to result in a goodness of fit within 10% of the optimal fit, this lends more credence to the notion that a minimal model could have *c*_A_=0. And, as previously argued, this means that a minimal model need not include the *A* equation at all.

### 3.3. Parameter Sloppiness Analysis of Ad/4-1BBL/IL-12 Model

To further explore the hypothesis that we can set *c*_A_=0 and therefore remove the variable *A* from our model, we conducted a sloppiness analysis of the Ad/4-1BBL/IL-12 model (Model 3c) in *c*_A_ − *c*_T_ − *c*_kill_ parameter space. We found that the Hessian matrix corresponding to the best-fit ellipsoid has the following eigenvalue-eigenvector pairs:

(12) $$\begin{array}{c} \lambda_{1}=19.85095,\\ {\vec{v}}_{1}=\left[\begin{array}{c} 0.000545\\ 0.708150\\ 0.706062 \end{array}\right], \end{array}$$

(13) $$\begin{array}{c} \lambda_{2}=0.09225,\\ {\vec{v}}_{2}=\left[\begin{array}{c} -0.003656\\ 0.706059\\ -0.708144 \end{array}\right],\\ {\vec{v}}_{3}=\left[\begin{array}{c} 0.999993\\ 0.002195\\ -0.002974 \end{array}\right]. \end{array}$$

(14) $$\begin{array}{c} \lambda_{3}=0.00000,\\ {\vec{v}}_{3}=\left[\begin{array}{c} 0.999993\\ 0.002195\\ -0.002974 \end{array}\right]. \end{array}$$

While the first two eigenvectors do not clearly point in the direction of one of the parameters, the final eigenvector clearly points along the axis of the first parameter (the *c*_A_ axis). Furthermore, the eigenvalue associated with this eigenvector is very small. Such a small eigenvalue means the length of the ellipsoid along the direction of that eigenvector is very large, and therefore, the *c*_A_ parameter can be classified as a soft parameter whose value has a minimal impact on the model dynamics.

### 3.4. A Minimal Model

Taken together, these analyses suggest that *c*_A_ can be fixed (at zero, as argued above), and therefore that the *A* variable can be removed from system (1)–(6) without sacrificing model fit to the data. If we remove the variable *A* from consideration, the parameters *c*_A_, *χ*_A_, and *δ*_A_ are no longer needed. This leaves us with the following *minimal model* which contains five variables (one less than the original) and eleven non-initial condition parameters (three less than the original):

(15) $$\begin{array}{c} \frac{dU}{dt}=rU-\beta \frac{UV}{N}-\left(\kappa_{0}+c_{\text{kill}}I\right)\frac{UT}{N}, \end{array}$$

(16) $$\begin{array}{c} \frac{dI}{dt}=\beta \frac{UV}{N}-\delta_{\text{I}}I-\left(\kappa_{0}+c_{\text{kill}}I\right)\frac{IT}{N}, \end{array}$$

(17) $$\begin{array}{c} \frac{dV}{dt}=u_{\text{V}}\left(t\right)+\alpha \delta_{\text{I}}I-\delta_{\text{V}}V, \end{array}$$

(18) $$\begin{array}{c} \frac{dT}{dt}=c_{\text{T}}I+\chi_{\text{D}}D-\delta_{\text{T}}T, \end{array}$$

(19) $$\begin{array}{c} \frac{dD}{dt}=u_{\text{D}}\left(t\right)-\delta_{\text{D}}D. \end{array}$$

When considering the treatment protocol Ad/4-1BBL/IL-12, the *c*_T_ parameter in this minimal model represents the joint impact that 4-1BBL and IL-12 have on recruiting tumor-targeted T cells. If we were considering only treatment with Ad/4-1BBL, *c*_T_ represents the singular impact 4-1BBL has, and if we were only considering treatment with Ad/IL-12, *c*_T_ represents the singular impact of IL-12 on T cell recruitment. The interpretation of all other parameters has not changed from the original model.

We have repeated the process of hierarchically finding the best-fit parameters for this minimal model, and its submodels corresponding to the different treatment protocols (Models 1–5, as detailed previously).  Table 2 gives the value of the best-fit parameters, along with the percent change in their values compared with the original model. These changes ranged from a 16% decrease to a 5% increase in the value of a single parameter (see Table 2).

**Figure 4:**
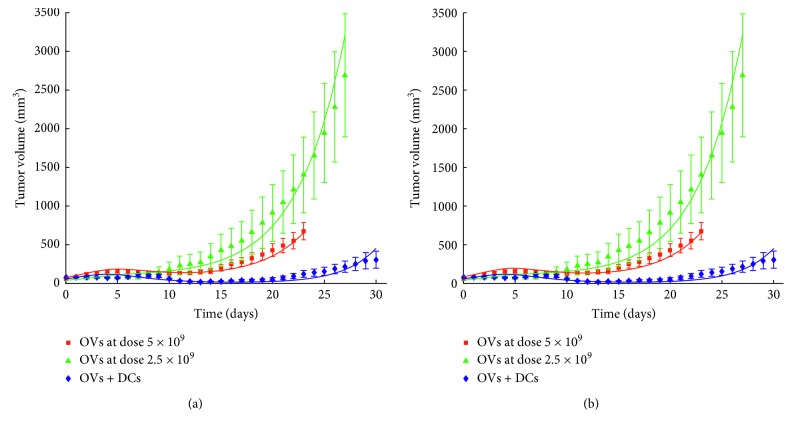
Experimental data from Huang et al. in which mice with B16-F10 subcutaneous tumors are intratumorally injected with different treatment protocols [4]. Data points represent mean tumor volume ± standard error in each group of 8-9 mice. All Ad/4-1BBL/IL-12 injections occur on days 0, 2, and 4, and DC injections, when given, occur on days 1, 3, and 5. Also shown in (a) are the best-fit solution curves from the original model presented in Equations (1)–(6) and in (b) are the best-fit solution curves from the minimal model presented in Equations (11)–(15).

Figure 4 illustrates how well the full model (Ad/4-1BBL/IL-12 and DCs) and submodel 3c (Ad/4-1BBL/IL-12, at two different doses) in their original and minimal forms fit the experimental data. Visually, the fits to the data from the minimal model (Figure 4) and the original model (Figure 4) are so similar they cannot be distinguished. Therefore, to quantify how the fit has changed from the original model to the minimal model, we compare the goodness-of-fit metric *ζ* in each case (see Table 2). Recall the goal of parameter fitting was to *minimizeζ* ; therefore, an increase in *ζ* means the model is a worse fit to the data, and a decrease in *ζ* means the model is a better fit to the data. We found that the goodness-of-fit metric (combined for both doses) went up about 3% (3% worse fit) for submodel 3c describing treatment with Ad/4-1BBL/IL-12. Surprisingly, and likely attributable to the random nature of the simulated annealing scheme used to fit the parameters (see [19] for details), the goodness-of-fit metric actually went down insignificantly (0.08% better fit) in the full model accounting for Ad/4-1BBL/IL-12 and DCs. This allows us to conclude that the minimal model describes the experimental data as well as the original model.

### 3.5. Is the Minimal Model Too Simple?

We have demonstrated that our minimal model is sufficient to describe the experimental data when tumors are treated with Ad/4-1BBL/IL-12 either in isolation or in combination with DC injections. Here, we explore the question of whether the model is too simple to describe the data. To begin, we turned to the model selection methods of Akaike information criterion (AIC) [32] and its variant AICc which corrects for small sample sizes [33], along with Bayesian information criterion (BIC) [34]. These can be used to evaluate different models and assign a numerical score to each model based on the goodness of fit to the data and the number of parameters in the model. This allows models based on different assumptions to be compared, with the aim of identifying the most plausible model [35]. To further validate our prediction that the minimal model is sufficient to describe the experimental data, we calculate the AIC, AICc, and BIC under the assumption that absolute model error is independent and normally distributed [35]:

(20) $$\begin{array}{c} \text{AIC}=n\ln\left(\frac{\zeta }{n}\right)+2k, \end{array}$$

(21) $$\begin{array}{c} \text{AICc}=n\ln\left(\frac{\zeta }{n}\right)+\frac{2nk}{n-k-1}, \end{array}$$

(22) $$\begin{array}{c} \text{BIC}=n\ln\left(\frac{\zeta }{n}\right)+k\ln\left(n\right), \end{array}$$

where *n*  is the number of time points for which we have data and *k* is the number of model parameters. Note we are using a modified version of these formulas to correspond with our goodness-of-fit function, *ζ*. All three criteria assign the lowest score, and therefore “select,” our minimal model of Ad/4-1BBL/IL-12 + DCs over the original model (see Table 3).

This information theoretic approach suggests that our minimal model is not too simple, when compared with the original model. However, it does not consider other components of the model that may make it too simplistic. To further investigate how much model complexity is needed to adequately describe the data, here we explore the impact of using a two-parameter growth model, the logistic equation (Equation (10)) to describe tumor growth without treatment. This is in comparison with the currently used one-parameter exponential growth term.

We approach this using a parameter sloppiness analysis. Since this model contains only two parameters, the analysis occurs in two-dimensional parameter space, which allows for nice visualizations of the results (see Figure 1). In particular, in Figure 1, we see all normalized points in *p*_1_ − *p*_2_ parameter space that give a goodness of fit within 10% of the optimal fit. In Figure 1, we can see the best-fit ellipsoid to this data, and in Figure 1, we see the eigenvectors of the Hessian associated with this ellipsoid.

We find that the tumor growth rate, *p*_1_, is a stiff parameter, as the eigenvector extending nearly along the *p*_1_ axis has relatively large eigenvalue of *λ* ≈ 5686. This means the value of *p*_1_ cannot deviate significantly from the optimal and still give a strong fit to the data. On the contrary, *p*_2_ is a soft parameter, as the eigenvector extending nearly along the *p*_2_ axis (distorted in figure due to scaling differences in the horizontal and vertical axis) has a small corresponding eigenvalue of *λ* ≈ 12. This means that the value of *p*_2_ can deviate significantly from its optimal value and still give a strong fit to the data. As *p*_2_ represents the carrying capacity in the logistic growth term, this says that the model for tumor growth is highly insensitive to the value of the carrying capacity. Since expanding the model to include logistic growth instead of exponential growth would introduce a soft parameter, we conclude that it is sufficient to use an exponential growth term to capture tumor behavior without treatment. The sufficiency of using an exponential growth term can be explained by revisiting the experimental data—the time scales for which tumor growth is considered are sufficiently short that the tumor is still in its near-exponential growth regime (even though its growth would eventually plateau). Hence, the model is highly insensitive to the choice of a carrying capacity, and the added complexity is not needed in this model.

### 3.6. Robustness Analysis

We have presented a variety of evidence that system (11)–(15) represents a minimally, but not too minimally, structured model describing treatment with immuno-enhanced OVs and dendritic cell injections. Here, we will create “virtual populations,” as indicated in the VEPART method, to classify various treatment protocols as either robust (effective in a large fraction of virtual populations) or fragile (ineffective in a large fraction of virtual populations). If the minimal model and original model yield similar predictions, this lends further support that the dynamics we are interested in capturing are adequately described by the minimal model.

The constraints imposed on this analysis were based on the experimental design in [4], and are as follows: (1) one treatment is administered per day, (2) there are six days of treatment, with three of the days being Ad/4-1BBL/IL-12, and three being DCs, and (3) the dose is fixed at the dose used in the experimental work [4], or a different fixed dose if specified. This results in twenty possible treatment protocols per fixed dose, and these protocols are then ranked from quickest time to tumor eradication (defined as tumor volume dropping below that of a single cell, estimated to be 10^−6^ mm^3^), to the largest volume after thirty days [19].

**Figure 5:**
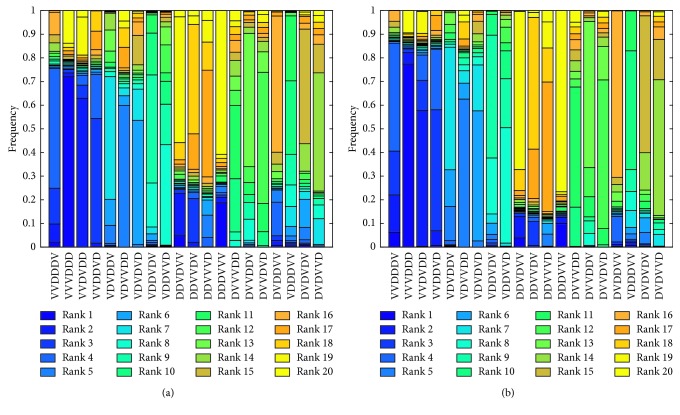
VEPART output at the experimental dose of Ad/4-1BBL/IL-12 and DCs used in Huang et al. [4]. The *x* axis indicates each of the 20 treatment protocols tested, with “V” representing Ad/4-1BBL/IL-12 treatment and “D” representing DC treatment on a given day. For each of the 20 treatment protocols, we see the frequency at which it ranks in positions 1 (best protocol) to 20 (worst protocol) for (a) the original model in system (1)–(6) and (b) the minimal model in system (11)–(15).

In our robustness analysis of the original model, the protocol of OV-OV-OV-DC-DC-DC was found to be optimal for the dose used in the experimental data. This was deemed the optimal protocol because it led to tumor eradication in the largest fraction of the 1000 virtual populations [19]. However, the response to this “optimal” protocol varied significantly across virtual populations, as shown in Figure 5(a). The “optimal” protocol was the best protocol to apply in 72.2% of the virtual populations, but was the worst protocol to apply in 13.8% of the virtual populations. Therefore, we previously classified the doses used in the experiments of Huang et al. [4] as *fragile*, since different virtual populations have a very different response to the same treatment protocol, including the one predicted to be “optimal.”

Here, we repeated the robustness analysis at the experimental dose used in Huang et al. [4] on the minimal model, and the results are shown in Figure 5(b). The protocol found to optimize treatment response in the minimal model is the same as in the original model, which is a good indication that the two models have similar dynamics. Furthermore, the “optimal” protocol was the best protocol to apply in 77.4% of the virtual populations (+5.2% from original model), but was the worst protocol to apply in 9.4% of the populations (−4.4% from original model). In other words, the optimal predicted at the experimental dose used in Huang et al. [4] is *fragile* whether or not we assess robustness using the original model or the minimal model.

The VEPART method was also applied to the original and minimal model in two regions of dosing space that differ from the experimental dose used in Huang et al. [4]. First, we considered increasing the Ad/4-1BBL/IL-12 dose by 50%, while simultaneously decreasing the DC dose by 50% (high OV/low DC). In the original model, the optimal at the high OV/low DC dose remained OV-OV-OV-DC-DC-DC [19], and this treatment proved to be more robust than the same treatment at the experimental dose. The same qualitative result holds when we consider the minimal model (data not shown).

**Figure 6:**
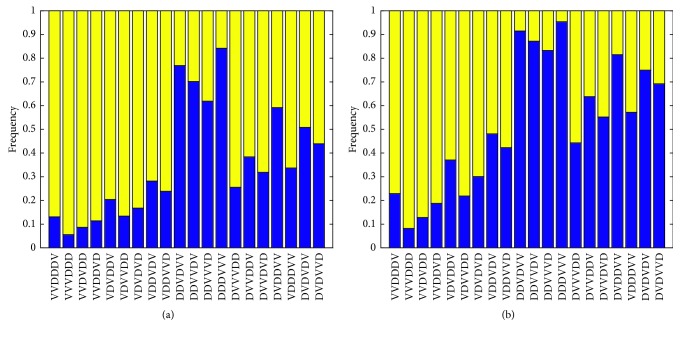
VEPART output at the low OV/high DC dose. The *x* axis indicates each of the 20 treatment protocols tested, with “V” representing Ad/4-1BBL/IL-12 treatment and “D” representing DC treatment on a given day. For each of the 20 treatment protocols, we see the frequency of virtual populations for which the specified treatment protocol leads to tumor eradication (blue) or tumor escape (yellow) for (a) the original model in system (1)–(6), and (b) the minimal model in system (11)–(15).

Next, we considered decreasing the Ad/4-1BBL/IL-12 dose by 50%, while simultaneously increasing the DC dose by 50% (low OV/high DC). In this case, the optimal treatment predicted by both the original and the minimal model is DC-DC-DC-OV-OV-OV. In the original model, this optimal proved to be the most robust of all, as it caused tumor eradication in a significant majority of the virtual populations (84.2%, see Figure 6(a)), and it ranked as the best protocol in 100% of the virtual populations [19]. In the minimal model, the optimal protocol still proves to be the most robust protocol considered, causing tumor eradication in 95.4% of the virtual populations (+11.2% from original model). And, just like for the original model, in the minimal model, the treatment ranks as the best protocol in 100% of the virtual populations.

Therefore, despite some quantitative changes, the main conclusion of our VEPART analysis remains unaltered whether we consider the original or the minimal model: the experimental dose used in Huang et al. [4] is still fragile (see Figure 5(b)), and we do not recommend treating at this region of dosing space. Instead, treatments should occur in the low OV/high DC region of dosing space, as the optimal protocol of DC-DC-DC-OV-OV-OV is predicted to be *robust* (see Figure 6). The similar nature of the predictions from the original and the minimal model lends further support to the sufficient nature of our minimal model.

## 4. Conclusions

In this study, we tackled a common challenge faced by mathematical biologists: identifying when one has developed a *minimal* model to describe an experimental dataset. Our approach combined the use of several methodologies, including an analysis of 95% credible intervals, a local sensitivity analysis, a global sensitivity analysis, a parameter sloppiness analysis, an information criteria analysis, and a comparison of model dynamics subject to a range of scenarios (treatment protocols). Applying these approaches to our model of oncolytic virus treatment in combination with dendritic cell injections led us to uncover that a reduced model, containing five (instead of six) variables and eleven (instead of fourteen) non-initial condition parameters, is sufficient and not too simplistic to describe the experimental data in [4].

Although the results presented herein are particular to one dataset and its corresponding model, these analyses can be applied in many other scenarios where it is of value to have an analytically tractable model with a minimal number of parameters. That said, this was not a comprehensive study of all aspects of the model. While we focused closely on robustness to perturbations in parameter values, we did not consider robustness to initial conditions, or to many of the functional forms used in the model, as has been considered elsewhere [13, 36–39]. Further, we did not study whether our ordinary differential equation model is structurally identifiable, meaning its parameters can be identified from perfect noise-free data, or if the model is practically identifiable, meaning the parameters can be identified in the case of imperfect, noisy data [40]. Despite not considering all possible ways to analyze the sufficiency of a mathematical model, we are able to simplify an existing mathematical model while providing evidence that the model is not too simple to describe important aspects of treatment with oncolytic viruses and dendritic cell injections.

While in many ways the approaches detailed herein run counter to the current trend of developing mechanistic models with significant biological detail, we believe that minimal models with sufficient complexity hold significant promise in the realm of *precision medicine* [20, 41, 42]. Precision/personalization raises a number of challenges, a significant one being the often sparse data available on an individual basis coupled with the high dimensionality of parameter space, even in a minimal model like the one proposed here. In future work, we will consider how global sensitivity analyses can help to identify the most important model parameters in a high-dimensional parameter space. This will allow us to leverage our minimal model to perform individualized fitting of mouse data, and to search for personalized optimal treatment protocols.

## Figures and Tables

**Table 1:** Best-fit parameters for the system in Equations (1)–(6) to the Ad/4-1BBL/IL-12 and DC data, along with the corresponding 95% credible interval, as computed in [19]. Also shown is the maximum extent to which each parameter value can deviate from its best-fit value and still give a fit within 10% of the optimal, as determined through a local sensitivity analysis [19].

Parameter	Best-fit	Maximum % deviation from best-fit value	95% credible interval

*r*	*r* ^*∗*^=0.3198	3%	[0.928*r*^*∗*^, 1.135*r*^*∗*^]
*β*	*β* ^*∗*^=0.00100854	0.7%	[0.939*β*^*∗*^, 1.050*β*^*∗*^]
*c*_A_	*c*_A_^*∗*^=0.000517	17,300%	[0.542*c*_A_^*∗*^, 3321*c*_A_^*∗*^]
*c*_T_	*c*_T_^*∗*^=1.6984	47.2%	[0.005*c*_T_^*∗*^, 2.445*c*_T_^*∗*^]
*c*_kill_	*c*_kill_^*∗*^=5.954 × 10^−7^	46.3%	[0.00017*c*_kill_^*∗*^, 1.895*c*_kill_^*∗*^]
*χ*_D_	*χ*_D_^*∗*^=4.6754	7%	[0.856*χ*_D_^*∗*^, 1.803*χ*_D_^*∗*^]

**Table 2:** Best-fit parameter values for the minimal model in Equations (11)–(15), along with the change in the goodness-of-fit metric *ζ* for the submodel of Ad/4-1BBL/IL-12 (Model 3c), and the full model for Ad/4-1BBL/IL-12 with DCs (Model 5). Also provided is the model number (which stage of the hierarchy) that was fit to arrive at the given parameter value. Once a value has been fit, it is used in all subsequent models.

Value	Best-fit (minimal model)	% deviation from original model	Model

*r*	$\hat{r}=0.3198$	0%	1
*β*	$\hat{\beta }=0.00100854$	0%	2
*c*_T_	${\hat{c}}_{\text{T}}=1.428064$	−15.92%	3c
*c*_kill_	${\hat{c}}_{\text{kill}}=6.234\times 10^{-7}$	+4.7%	3
*χ*_D_	${\hat{\chi }}_{\text{D}}=4.901894$	+4.84%	4 and 5

Value	% change in *ζ* from original model		

*ζ*_*Ad*/4−1*BBL*/*IL*−12_	+3.05%		
*ζ*_*Ad*/4−1*BBL*/*IL*−12+*DC*_	−0.08%		

**Table 3:** Values of AIC, AICc, and BIC for both the original and minimal model of Ad/4-1BBL/IL-12 + DCs.

	Original model	Minimal model
AIC	325.3096	319.3474
AICc	350.6029	339.2206
BIC	339.3096	327.3474

## Data Availability

Previously reported murine tumor volume data were used to support this study. These prior studies (and datasets) are cited at relevant places within the text as reference [4].
